# The shared risk of diabetes between dog and cat owners and their pets: register based cohort study

**DOI:** 10.1136/bmj.m4337

**Published:** 2020-12-10

**Authors:** Rachel Ann Delicano, Ulf Hammar, Agneta Egenvall, Carri Westgarth, Mwenya Mubanga, Liisa Byberg, Tove Fall, Beatrice Kennedy

**Affiliations:** 1 Department of Medical Sciences, Molecular Epidemiology and Science for Life Laboratory, Uppsala University, 751 85 Uppsala, Sweden; 2 Department of Clinical Sciences, Swedish University of Agricultural Sciences, Uppsala, Sweden; 3 Institute of Infection, Veterinary and Ecological Sciences, University of Liverpool, Liverpool, UK; 4 Department of Medical Epidemiology and Biostatistics, Karolinska Institutet, Stockholm, Sweden; 5 Department of Surgical Sciences, Orthopaedics, Uppsala University, Uppsala, Sweden

## Abstract

**Objective:**

To investigate whether dog and cat owners and their pets share a risk of developing diabetes.

**Design:**

Cohort study.

**Setting:**

Register based longitudinal study, Sweden.

**Participants:**

208 980 owner-dog pairs and 123 566 owner-cat pairs identified during a baseline assessment period (1 January 2004 to 31 December 2006).

**Main outcome measures:**

Type 2 diabetes events in dog and cat owners and diabetes events in their pets, including date of diagnosis during the follow-up period (1 January 2007 to 31 December 2012). Owners with type 2 diabetes were identified by combining information from the National Patient Register, the Cause of Death Register, and the Swedish Prescribed Drug Register. Information on diabetes in the pets was extracted from veterinary care insurance data. Multi-state models were used to assess the hazard ratios with 95% confidence intervals and to adjust for possible shared risk factors, including personal and socioeconomic circumstances.

**Results:**

The incidence of type 2 diabetes during follow-up was 7.7 cases per 1000 person years at risk in dog owners and 7.9 cases per 1000 person years at risk in cat owners. The incidence of diabetes in the pets was 1.3 cases per 1000 dog years at risk and 2.2 cases per 1000 cat years at risk. The crude hazard ratio for type 2 diabetes in owners of a dog with diabetes compared with owners of a dog without diabetes was 1.38 (95% confidence interval 1.10 to 1.74), with a multivariable adjusted hazard ratio of 1.32 (1.04 to 1.68). Having an owner with type 2 diabetes was associated with an increased hazard of diabetes in the dog (crude hazard ratio 1.28, 1.01 to 1.63), which was attenuated after adjusting for owner’s age, with the confidence interval crossing the null (1.11, 0.87 to 1.42). No association was found between type 2 diabetes in cat owners and diabetes in their cats (crude hazard ratio 0.99, 0.74 to 1.34, and 1.00, 0.78 to 1.28, respectively).

**Conclusions:**

Data indicated that owners of a dog with diabetes were more likely to develop type 2 diabetes during follow-up than owners of a dog without diabetes. It is possible that dogs with diabetes could serve as a sentinel for shared diabetogenic health behaviours and environmental exposures.

## Introduction

Type 2 diabetes is recognised as a major global public health challenge, with more than 400 million individuals affected worldwide.^1^ In Sweden, the point prevalence of type 2 diabetes in adults almost reaches 5%,^2^ and this prevalence is projected to increase in the coming decades. The expected increase is partly attributable to the population aging, and it is also driven by profound shifts in lifestyle behaviours and obesity rates.^3^ The prevalence of diabetes in dogs and cats also might be on the increase.^4^ ^5^

Dogs and cats that develop diabetes usually present with distinct and rapidly progressing symptoms, including excessive thirst, polyuria, and weight loss, similar to the clinical presentation of type 1 diabetes in humans.^6^ The pathogenesis of diabetes in dogs is heterogeneous, and the cause of hyperglycaemia can be categorised as primary insulin deficiency diabetes or insulin resistant diabetes.^7^ Insulin resistant diabetes secondary to diabetogenic hormone changes during the prolonged dioestrus phase is of particular relevance in a Swedish context, as spaying of female dogs is rare and often performed late in life for medical reasons.^8^ In contrast, the British Veterinary Association recommends the neutering of all pet dogs not intended for breeding,^9^ and most female dogs in the UK are spayed.

The pathogenesis of diabetes in cats is suggested to largely correspond to that of type 2 diabetes in humans,^10^ with reduced insulin sensitivity as a key feature. Established non-modifiable risk factors for diabetes in both dogs and cats include age, sex, and breed.^11^ ^12^ Corresponding with typical risk factors for type 2 diabetes in humans, however, diet, obesity, and level of physical activity influence the risk of diabetes in both dogs and cats.^13^ ^14^ ^15^ ^16^

Cross sectional studies have indicated an association between adiposity in dog owners and their pets,^17^ ^18^ suggesting that the two might share health behaviours, including activity level, that could affect their morbidity alike. This finding matches that of studies on diabetes in spouses, which reported that spouses of individuals with a diagnosis of type 2 diabetes might be at increased risk of developing diabetes themselves,^19^ ^20^ likely as a result of shared lifestyle factors and adiposity rates as well as shared socioeconomic circumstances. Furthermore, owners and pets living in the same household could be similarly exposed to environmental diabetogenic factors, including pollutants^21^ ^22^ and endocrine disrupting chemicals.^23^ However, no studies have been performed on the shared risk of diabetes between dog and cat owners and their pets.

We aimed to contribute to the knowledge base on the potential interplay between human and pet health as outlined in One Health initiatives.^24^ A household might be viewed as a functional unit, with a large potential interdependence in lifestyle behaviours and disease risks owing to the human-animal bond between owners and their pets. Linking data from Swedish national population and health registers with information from the largest pet insurance company in Sweden, we investigated whether dog and cat owners and their pets share the risk of diabetes.

## Methods

### Study population

Agria Pet Insurance is the largest animal insurance company in Sweden and covers an estimated 40% of the dog population^25^ and 23% of the cat population.^26^ ^27^ The study population was generated by register linkage between information from Agria Pet Insurance and official Swedish registers (held by Statistics Sweden and the Swedish Board of Health and Welfare) using the owner’s unique 10 digit national identification number assigned to all residents in Sweden.^28^ We received only pseudonymised data.

We identified 151 054 dog owners and 74 336 cat owners born before 1961 with an active veterinary care policy with Agria Pet Insurance at any time from 1 January 2004 to 31 December 2006 (the baseline assessment period). The cut-off at 1961 in the initial register linkage was chosen to exclude younger individuals who were at lower risk of type 2 diabetes. From the Register of the Total Population we further extracted information on 94 327 spouses or cohabiting partners of the dog owners and 41 764 spouses or cohabiting partners of the cat owners. For non-married partners, information was only available for cohabiting partners who had children in common. The spouses and cohabiting partners were also considered to be pet owners, and the term pet owner used hereafter refers to both.

We excluded 2958 dog owners and 1192 cat owners who died or emigrated from Sweden before 1 January 2007, and 5306 additional dog owners and 2428 cat owners with incomplete baseline or follow-up data or unclear emigration status. After these exclusions we had 237 117 dog and 112 511 cat owners. We then included 218 392 eligible insured dogs and 122 063 eligible insured cats and identified all owner-dog pairs and owner-cat pairs in our study population.

**Fig 1:**
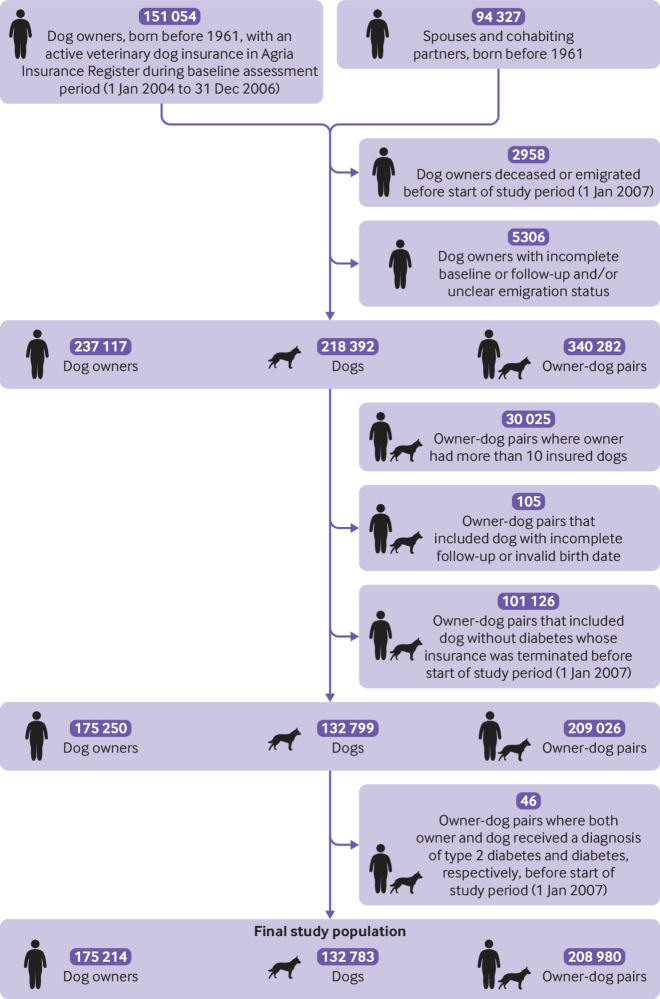
Flowchart of owner-dog study population

**Fig 2:**
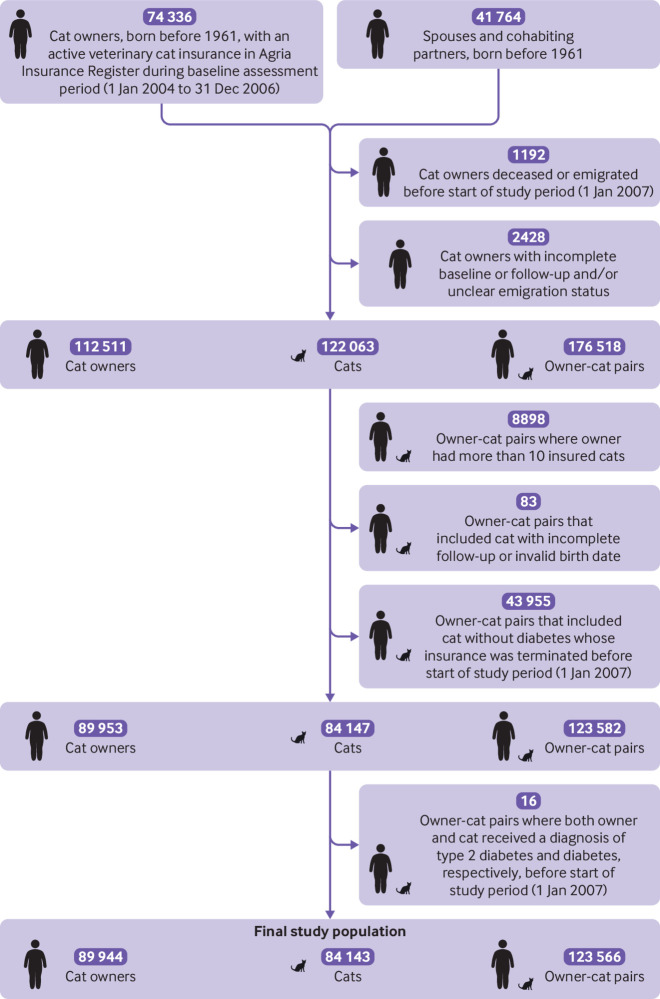
Flowchart of owner-cat study population

Of these owner-pet pairs, we excluded 30 025 owner-dog pairs and 8898 owner-cat pairs because the owners each had more than 10 insured pets and probably represent breeders where not all pets are kept within the household of the registered owner. We also excluded 105 owner-dog pairs and 83 owner-cat pairs with incomplete pet follow-up or an invalid pet birth date, as well as 101 126 owner-dog pairs and 43 955 owner-cat pairs that included a pet without diabetes whose insurance was terminated before 1 January 2007. Lastly, we excluded 46 owner-dog pairs and 16 owner-cat pairs when both owner and pet had a diagnosis of diabetes before 1 January 2007. The final study population comprised 208 980 owner-dog pairs and 123 566 owner-cat pairs (fig 1 and fig 2).

If an individual owned both a dog and a cat, we included that individual as both a dog owner and a cat owner, and together with the pets these formed both an owner-dog pair and an owner-cat pair. Similarly, if an individual owned multiple dogs and cats, that individual formed one owner-pet pair for each pet.

### Diabetes assessment

Owners were considered to have type 2 diabetes if they had a main or secondary diagnosis (international classification of diseases and related health problems, 10th revision) of type 2 diabetes (ICD-10 code E11) within the National Patient Register (inpatient and outpatient specialist care) or in the Cause of Death Register, or both. In addition, we considered owners to have type 2 diabetes if they had at least one dispensed prescription of an oral diabetes drug or a non-insulin injectable diabetes drug, or both (Anatomic Therapeutic Codes A10B and A10X) in the Swedish Prescribed Drug Register, even without registration in the other registers. These data were available from 1 July 2005. Type 1 diabetes diagnoses or insulin prescriptions were not included in our study. Information on diabetes status of the pets was extracted from the Agria Pet Insurance data. In Sweden, veterinarians report disease diagnoses to this register using a standardised coding system.^29^ We defined pets as having diabetes in association with claims for any of the following codes: EA234 (diabetes), EA2341 (diabetes without complications), EA2342 (diabetes with complications), and EA23421 (diabetes with ketoacidosis). For both owners and pets, we considered the first record indicating a diabetes diagnosis as the day of diagnosis.

### Participant characteristics

#### Owners

From the Total Population Register, we retrieved information on country of birth (categorised as Sweden, another Nordic country (Norway, Denmark, Iceland, Finland, Åland, and the Faroe Islands), or a non-Nordic country) and on Swedish region of residence (south: Götaland, middle: Svealand, or north: Norrland). Baseline household socioeconomic circumstances in 2006 were extracted from the Longitudinal Integration Database for Health Insurance and Labour Market Studies,^30^ including highest attained education level (categorised as compulsory education only, secondary education, or university education), cohabitation status (categorised as married or cohabiting, or not married or cohabiting), population density, and income. Information on education level was only available for individuals born from 1926. Population density was defined as the number of inhabitants per square kilometre in the home municipality, whereas income represented the individual annual disposable income in Swedish kroner. Both population density and income had skewed distributions, and therefore we log transformed density and divided income into 10ths in the analyses.

#### Pets

Information on date of birth, date of start and end of insurance, breed, and date of death of the pets were available from the Agria Pet Insurance data. We categorised the dog and cat breeds as those with high, moderate, or low risk of diabetes in accordance with two previous Swedish large cohort studies that investigated diabetes incidence in dog^11^ and cat^12^ breeds (supplementary tables 1 and 2).

### Statistical analysis

**Fig 3:**
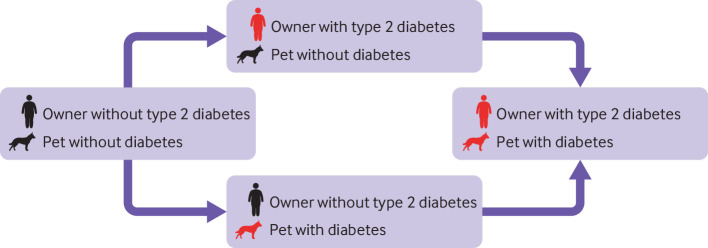
Weibull-Markov multistate model. Owner-pet pairs were classified according to combined diabetes status during the baseline assessment period (1 January 2004 to 31 December 2006). During follow-up (1 January 2007 to 31 December 2012) all owner-pet pairs were monitored for a new diabetes diagnosis in either owner or pet, which constituted a transition between states. Hazard ratios for type 2 diabetes in owners and diabetes in pets were calculated comparing the transition rate from pet with diabetes to diabetes in owner and pet with that of transition from no diabetes in owner or pet to owner with type 2 diabetes, and comparing the transition rate from owner with type 2 diabetes to diabetes in both owner and pet with that of transition from no diabetes in owner or pet to diabetes in pet

We utilised a Weibull-Markov multistate model^31^ in which the combined diabetes status of the owner-pet pairs during baseline determined their diagnoses in relation to diabetes at the end of the baseline assessment period (1 January 2004 to 31 December 2006). Any transitions between diagnoses were monitored during follow-up (1 January 2007 to 31 December 2012; fig 3). In accordance with the strengthening the reporting of observational studies in epidemiology guidelines, we did not do any power calculations before analyses as the study was performed in a fixed available sample.^32^

For each owner-pet pair, we defined the combined baseline status as no diabetes in owner or pet, owner with type 2 diabetes, diabetes in pet only, and diabetes in owner and pet. Owner-pet pairs that shared a diagnosis of diabetes during baseline were not assessed during follow-up and were excluded from analyses as they could make no further transition. During the longitudinal follow-up period, we defined a transition as when the owner or the pet received a diagnosis of type 2 diabetes or diabetes, respectively.

After the start of follow-up period, we censored owner-pet pairs at death of the owner or pet, emigration of the owner, termination of pet insurance, or end of follow-up (31 December 2012), whichever occurred first. However, we did not censor owner-pet pairs due to death of the pet if the pet had already had a diagnosis of diabetes since any further transition would only be due to change in the type 2 diabetes status of the owner.

A total of four transitions were possible (see figure 3): no diabetes in owner or pet to owner with type 2 diabetes; no diabetes in owner or pet to diabetes in pet; owner with type 2 diabetes to diabetes in both owner and pet; and diabetes in pet only to diabetes in both owner and pet. Hazard ratios were calculated for each transition. To investigate whether the hazard ratios differed according to diabetes status of the other part of the owner-pet pair, we investigated whether the hazard ratios in our Weibull-Markov model (comparing transition rates: from diabetes in pet to diabetes in both owner and pet with those from no diabetes in owner or pet to owner with type 2 diabetes, and from owner with type 2 diabetes to diabetes in both owner and pet with no diabetes in owner or pet to diabetes in pet) were significant at an α level of 0.05. In the main analyses, we present a crude unadjusted model and a fully adjusted model (adjusted for age and sex of owner, age and sex of pet, breed group, and personal and socioeconomic characteristics of owner, including country of birth, population density, region of residence, highest attained education level, marital status, and disposable income). In the supplementary file we have included information on the attenuations of the estimates for owner-dog pairs and owner-cat pairs when the covariates are added consecutively. In those analyses, model 1 constituted the crude non-adjusted model. Models 2a and 2b were adjusted for the age and sex of the owner and pet, with certain constraints depending on the transition. Model 3 was adjusted for age and sex of the owner and pet, without constraints. To model 3, we sequentially added breed group (model 4), country of birth (model 5), population density and region of residence (model 6), and highest attained education level, marital status, and disposable income (model 7, corresponding to our fully adjusted model). In all analyses, breed group, country of birth, education level, and region of residence were modelled as categorical variables. Income group was modelled as a continuous variable ranging from 1 to 10, with the first decile corresponding to the value 1, the second decile to the value 2, and so on. Age of owner and age of pet were modelled using restricted cubic splines, with knots placed on the 10th, 50th, and 90th centiles. Owner-dog and owner-cat pairs were analysed separately in all analyses, and cluster robust standard errors were used to account for the dependence of owners and pets within households.^33^ Since human covariates might exert a stronger influence on incidence of type 2 diabetes in humans (transitions from diabetes in pet to diabetes in both owner and pet and from no diabetes in owner or pet to owner with type 2 diabetes) and pet covariates on incidence of diabetes in pets (transitions from owner with type 2 diabetes to diabetes in both owner and pet and from no diabetes in owner or pet to diabetes in pet), all models included an interaction term between each covariate and the transition with the constraint that the effect of the covariate should be equal within transitions from diabetes in pet to diabetes in both owner and pet and from no diabetes in owner or pet to owner with type 2 diabetes, and transitions from owner with type 2 diabetes to diabetes in both owner and pet and from no diabetes in owner or pet to diabetes in pet. Furthermore, in model 2a, the effect of the pet’s age and sex on transitions from diabetes in pet to diabetes in both owner and pet and from no diabetes in owner or pet to owner with type 2 diabetes (owner type 2 diabetes rates) was set to 0, as was the effect of the owner’s age and sex on transitions from owner with type 2 diabetes to diabetes in both owner and pet and from no diabetes in owner or pet to diabetes in pet (pet diabetes rates). In model 2b, the effect of the pet’s sex on transitions from diabetes in pet to diabetes in both owner and pet and from no diabetes in both owner and pet to owner with type 2 diabetes was set to 0, and the effect of the owner’s sex on transitions from owner with type 2 diabetes to diabetes in both owner and pet and from no diabetes in owner or pet to diabetes in pet was set to 0.

### Sensitivity analyses

The hazard ratio of diabetes in dogs was attenuated when age of owner was included as a covariate in model 2b (see supplementary file). Supplementary figure 3 shows the association between age of dog owners on hazard of diabetes in their dogs.

We further aimed to assess potential bias from the decision to include owner-pet pairs with dead pets with a diagnosis of diabetes. We therefore also performed a sensitivity analysis in which we excluded all pets who died before the start of the follow-up period, regardless of their diabetes status, as well as follow-up time contributed by pet-owner pairs after the death of a pet with a diagnosis of diabetes.

All analyses were performed in Stata/MP 14 (StataCorp, TX).

### Patient and public involvement

Neither study participants nor the public were in any way involved in the design, conduct, reporting, or planning of dissemination of our research.

## Results

### Baseline characteristics of owner-dog pairs

The owner-dog study population included 208 980 pairs, comprising 175 214 owners and 132 783 pets (fig 1 and table 1). Most of the pairs (n=197 795, 94.6%) did not have diabetes at the start of follow-up. Compared with dog owners without type 2 diabetes, dog owners with type 2 diabetes were older, more often men, and less likely to have a university level education or to be married or cohabitating. Owners in pairs where only the dog had diabetes at the start of follow-up had the lowest median income among the transition types. Dogs in these owner-dog pairs were on average older, more often female, and more often belonged to breeds with a high risk of diabetes than dogs in owner-dog pairs with no diabetes or with an owner with type 2 diabetes.

**Table 1:** Baseline characteristics of dog owners and their pets at start of study period, 1 January 2007. Values are numbers (percentages) unless stated otherwise

Characteristics	Total	Owner and dog without diabetes	Owner with type 2 diabetes, dog without diabetes	Owner without type 2 diabetes, dog with diabetes
Owner-dog pairs*	208 980	197 795 (94.6)	10 393 (5.0)	792 (0.4)
**Dog owners**				
No in sample	175 214	165 863	8831	788
Median (interquartile range) age (years)	57 (51-63)	56 (51-63)	62 (57-68)	59 (52-66)
Women	89 029 (50.8)	85 426 (51.5)	3325 (37.7)	412 (52.3)
Men	86 185 (49.2)	80 437 (48.5)	5506 (62.3)	376 (47.7)
Country of birth:				
Sweden	163 372 (93.2)	154 750 (93.3)	8144 (92.2)	731 (92.8)
Other Nordic countries†	6763 (3.9)	6323 (3.8)	417 (4.7)	34 (4.3)
Non-Nordic countries	5079 (2.9)	4790 (2.9)	270 (3.1)	23 (2.9)
Median (interquartile range) population density‡	561 (218-1207)	572 (218-1207)	477 (207-1095)	519 (193-1207)
Region of residence:				
Götaland	88 853 (50.7)	84 127 (50.7)	4477 (50.7)	365 (46.3)
Svealand	63 358 (36.2)	59 961 (36.2)	3192 (36.1)	331 (42.0)
Norrland	23 003 (13.1)	21 775 (13.1)	1162 (13.2)	92 (11.7)
Education level:				
Compulsory	44 627 (25.5)	41 123 (24.8)	3346 (37.9)	231 (29.3)
Secondary	79 673 (45.5)	75 639 (45.6)	3813 (43.2)	344 (43.7)
University	50 914 (29.1)	49 101 (29.6)	1672 (18.9)	213 (27.0)
Marital status:				
Married or cohabiting	141 606 (80.8)	134 224 (80.9)	6977 (79.0)	629 (79.8)
Not married or cohabiting	33 608 (19.2)	31 639 (19.1)	1854 (21.0)	159 (20.2)
Median (interquartile range) disposable income§	1628 (1152-2285)	1631 (1155-2289)	1584 (1113-2213)	1551.5 (1111.5-2239)
**Dogs**				
No in sample	132 783	129 348	10 052	517
Median (interquartile range) age (years)	5.4 (2.7-8.6))	5.4 (2.6-8.6)	5.7 (2.8-8.8)	11.7 (9.7-13.4)
Female	67 785 (51.0)	65 911 (51.0)	5157 (51.3)	383 (74.1)
Male	64 998 (49.0)	63 437 (49.0)	4895 (48.7)	134 (25.9)
Breed groups:				
High diabetes risk¶	14 766 (11.1)	14 247 (11.0)	1222 (12.2)	164 (31.7)
Low diabetes risk**	22 711 (17.1)	22 151 (17.1)	1752 (17.4)	22 (4.3)
Moderate diabetes risk††	95 306 (71.8)	92 950 (71.9)	7078 (70.4)	331 (64.0)

SEK 1.00 (£0.01; $0.12; €0.10).

* Row percentages

† Norway, Denmark, Iceland, Finland, Åland, and the Faroe Islands.

‡ Number of inhabitants per square kilometre in the home municipality.

§ Individual disposable income in thousands (SEK) annually.

¶ Australian Terrier, Samoyed, Swedish Lapphund, Swedish elkhound, border collie, Finnish hound, drever, west Highland white terrier, Hamilton hound, and poodle (miniature and toy).

** Jack Russell terrier, miniature dachshund, German shepherd, rough haired collie, standard poodle, soft coated wheaten terrier, bearded collie, golden retriever, boxer, and papillon.

†† All other pure breeds as well as crossbreed or mixed breed dogs, or both.

### Baseline characteristics of owner-cat pairs

The owner-cat study population included 123 566 owner-cat pairs, consisting of 89 944 cat owners and 84 143 cats (fig 2 and table 2). Most of the pairs (n=117 391, 95.0%) had no diabetes at the start of the follow-up. Compared with cat owners without type 2 diabetes, cat owners with type 2 diabetes were on average older, more often men, and less likely to have a university level education. Owners in owner-cat pairs where only the cat had diabetes at the start of follow-up were more likely to be women and not cohabiting and less likely to live in Norrland or an area with a low population density compared with owners and cats without diabetes or owner with type 2 diabetes. Cat owners without type 2 diabetes but with a pet with diabetes also had the highest median income. In owner-cat pairs where only the pet had diabetes, the cats were older, more often male, and belonged to breeds with a high risk of diabetes more often than the owner-cat pairs in which owner and pet did not have diabetes or the owner had type 2 diabetes.

**Table 2:** Baseline characteristics of cat owners and their pets at start of study period, 1 January 2007. Values are numbers (percentages) unless stated otherwise

Characteristics	Total	Owner and cat without diabetes	Owner with type 2 diabetes, cat without diabetes	Owner without type 2 diabetes, cat with diabetes
Owner-cat pairs*	123 566	117 391 (95.0)	5622 (4.5)	553 (0.4)
**Cat owners**				
No in sample	89 944	85 458	4179	541
Median (interquartile range) age (years)	55 (50-61)	55 (50-61)	61 (55-67)	57 (52-62)
Women	50 940 (56.6)	48 886 (57.2)	1860 (44.5)	340 (62.8)
Men	39 004 (43.4)	36 572 (42.8)	2319 (55.5)	201 (37.2)
Country of birth:				
Sweden	82 446 (91.7)	78 411 (91.8)	3751 (89.8)	497 (91.9)
Other Nordic countries†	3961 (4.4)	3714 (4.3)	239 (5.7)	17 (3.1)
Non-Nordic countries	3537 (3.9)	3333 (3.9)	189 (4.5)	27 (5.0)
Median (interquartile range) population density‡	867 (357-4720)	872 (358-4720)	836 (325-3564)	1219 (519-10 866)
Region of residence:				
Götaland	41 885 (46.6)	39 821 (46.6)	1922 (46.0)	235 (43.4)
Svealand	41 900 (46.6)	39 826 (46.6)	1922 (46.0)	282 (52.1)
Norrland	6159 (6.8)	5811 (6.8)	335 (8.0)	24 (4.4)
Education level:				
Compulsory	18 792 (20.9)	17 276 (20.2)	1447 (34.6)	109 (20.1)
Secondary	40 829 (45.4)	38 813 (45.4)	1877 (44.9)	262 (48.4)
University	30 323 (33.7)	29 369 (34.4)	855 (20.5)	170 (31.4)
Marital status:				
Married or cohabiting	65 818 (73.2)	62 609 (73.3)	3013 (72.1)	351 (64.9)
Not married or cohabiting	24 126 (26.8)	22 849 (26.7)	1166 (27.9)	190 (35.1)
Median (interquartile range) disposable income§	1688 (1221-2321)	1694 (1224-2326)	1578 (1159-2194)	1788 (1261-2473)
**Cats**				
No in sample	84 143	81 694	5440	394
Median (interquartile range) age (years)	5.6 (3.0-9.0)	5.6 (3.0-8.9)	5.6 (3.0-9.0)	13.5 (11.1-15.6)
Female	39 067 (46.4)	38 019 (46.5)	2461 (45.2)	115 (29.2)
Male	45 076 (53.6)	43 675 (53.5)	2979 (54.8)	279 (70.8)
Breed groups:				
High diabetes risk¶	6994 (8.3)	6743 (8.3)	464 (8.5)	69 (17.5)
Low diabetes risk**	10 269 (12.2)	10 019 (12.3)	634 (11.7)	25 (6.3)
Moderate diabetes risk††	66 880 (79.5)	64 932 (79.5)	4342 (79.8)	300 (76.1)

SEK 1.00 (£0.01; $0.12; €0.10).

* Row percentages

† Norway, Denmark, Iceland, Finland, Åland, and the Faroe Islands.

‡ Number of inhabitants per square kilometre in the home municipality.

§ Individual disposable income in thousands (Swedish kroner) annually.

¶ Burmese, Russian blue, Norwegian forest cat, and European shorthair.

** Maine coon, Persian/exotic, British shorthair, Siberian, Birman, ragdoll, and Bengal.

†† All other pure breeds as well as domestic cats.

### Diabetes in dog owners and dogs

During a maximum of six years (median 3.4 years) of follow-up, the observed incidence rate of type 2 diabetes in dog owners was 7.7 cases per 1000 person years at risk and in dogs was 1.3 cases per 1000 dog years at risk.

**Fig 4:**
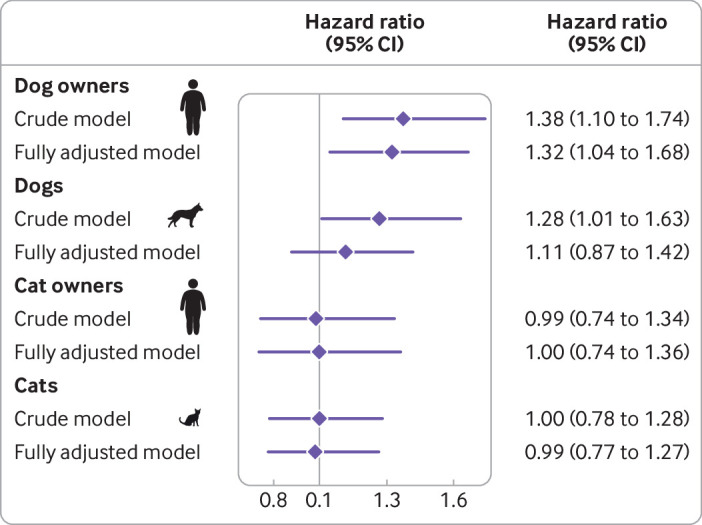
Hazard ratios and 95% confidence intervals for type 2 diabetes in dog and cat owners and diabetes their pets during follow-up from 1 January 2007 to 31 December 2012 (n=211 914 owner-dog pairs and n=123 566 owner-cat pairs). Fully adjusted models adjusted for age and sex of owner, age and sex of pet, breed group, and personal and socioeconomic characteristics of the owner, including country of birth, population density, region of residence, highest attained education level, marital status, and disposable income

Compared with owning a dog without diabetes, owning a dog with diabetes was associated with an increased hazard of type 2 diabetes (crude model: hazard ratio 1.38, 95% confidence interval 1.10 to 1.74, fig 4 and supplementary figure 1). The estimate did not change noticeably after adjusting for all additional available covariates (fully adjusted model: 1.32, 1.04 to 1.68).

In the crude model, the hazard of developing diabetes was found to be higher in dogs with an owner who had type 2 diabetes compared with dogs with an owner who did not have type 2 diabetes (hazard ratio 1.28, 95% confidence interval 1.01 to 1.63). This estimate, however, was attenuated in the fully adjusted model, with the confidence interval crossing the null (1.11, 0.87 to 1.42). In additional analyses the main attenuation was observed when age of the owner was introduced as a covariate (model 2B, 1.15, 0.90 to 1.46, supplementary figure 1), as illustrated in supplementary figure 2.

### Diabetes in cat owners and cats

During a maximum of six years (median 3.8 years) of follow-up, the observed incidence rate of type 2 diabetes in cat owners was 7.9 cases per 1000 person years at risk and in cats was 2.2 cases per 1000 cat years at risk.

Compared with owning a cat without diabetes, owning a cat with diabetes was not associated with an increased risk of type 2 diabetes (crude model: 0.99, 0.74 to 1.34, fully adjusted model: 1.00, 0.74 to 1.36, fig 4 and supplementary figure 3). Similarly, the risk of diabetes was not observed to increase in cats with an owner with type 2 diabetes (crude model: 1.00, 0.78 to 1.28, fully adjusted model: 0.99, 0.77 to 1.27).

### Sensitivity analyses

The study population size in which both owners and their pets had diabetes was reduced after excluding owner-pet pairs in which the pet had died during baseline or follow-up, regardless of the pet’s diabetes status. The estimates from these sensitivity analyses closely resembled those of the main analyses although with wider confidence intervals (supplementary figures 4 and 5).

## Discussion

In this large cohort study, we found that ownership of a dog with diabetes was associated with an increased hazard of type 2 diabetes in the dog owner. The estimate was not attenuated when available shared risk factors were considered. We detected no association between type 2 diabetes in cat owners and diabetes in their cats.

### Strengths and limitations of this study

Strengths of this study include the population based prospective study design, unique data linkage, and essentially complete follow-up. Some potential limitations apply. Firstly, information on health behaviours such as diet and physical activity level were not available, preventing investigation as to whether these represented the underlying causes of the associations. Secondly, owners with type 2 diabetes, or owners of a dog with diabetes, might have an increased awareness of overt diabetes symptoms and thus contribute to a surveillance bias effect within the owner-dog pair. Dogs with diabetes exhibit a distinct and rapidly progressive symptomatology, and subclinical diabetes or a pre-diabetic state is not commonly identified in dogs, not even in breeds with a high risk of diabetes.^11^ ^34^ ^35^ ^36^ It is therefore unlikely that any increase in the hazard of diabetes in dogs associated with an owner with type 2 diabetes is a result of intensified veterinary screening. In contrast, dog owners who are aware of diabetes as a disease because of their dog’s diabetes might be more likely to request laboratory screening from their doctor even with little or no symptoms of hyperglycaemia, leading to an increased early detection rate of type 2 diabetes in owners. Thirdly, we were not able to identify individuals with type 2 diabetes who do not receive drug treatment, which has been estimated at about 25% of the patients with type 2 diabetes in primary care.^37^ As for diabetes diagnoses in pets, there is little risk of misclassification of disease, as the clinical presentation is usually straightforward and the diagnostic procedures simple. However, insurance claims that do not meet the deductible would often not be submitted and those cases will not have been detected. Furthermore, dogs of crossbreed or mixed breed and dogs older than 10 years might be underrepresented in the insurance database,^8^ and our findings might not apply to these owner-dog pairs. The insurance coverage of the Swedish cat population is lower, and it is not known if insured cats are typical of all cats in Sweden,^12^ potentially limiting our external validity on diabetes incidence in other owner-cat-pairs. In addition, the maximum follow-up time in our study was six years, and therefore any diagnosed type 2 diabetes in the owner or diabetes in the pet after that time will not be included in our analyses. Lastly, our study population consisted of pet owners who had the financial means to take out veterinary insurance. The overall generalisability of our findings might therefore not extend to pet owners who, for financial or other reasons, decline pet health insurance. Similarly, it is possible that generalisability might only apply to other countries with similar regulations and practices for pet ownership.^38^

### Comparison with other studies

Several different underlying mechanisms might explain the observed association between type 2 diabetes in dog owners and risk of diabetes in their pet. Firstly, dog owners and their dogs could share lifestyle behaviours that affect the risk of diabetes. Cross sectional studies in veterinary clinics have reported an association between owner and dog adiposity.^17^ ^18^ A larger European cross sectional study based on online questionnaires, however, did not find such an association,^39^ although that study could have been limited by the owners’ assessment of the body composition of their dog. Dietary habits of the dog owners might also influence their pets’ diet and risk of adiposity—for example through portion control, frequency of feedings, and whether owners provide table scraps in addition to dog food.^15^ ^16^ ^40^ ^41^ The use and timing of dog treats has been associated with the weight of owners,^17^ suggesting an interplay between weight of a dog owner and energy intake in the pet. Although it has been assumed that diabetes in dogs has an autoimmune origin because of the insulinopenic presentation, other studies emphasise the heterogeneity of the pathogenesis of diabetes in dogs^7^ and have identified obesity as a risk factor for the development of diabetes.^15^ Being overweight is also associated with an increased risk of diabetes in Swedish and Norwegian elkhounds.^42^ Intact females of these two breeds have a predisposition for progesterone related diabetes,^42^ suggesting additive effects of hereditary diabetes risk and body composition. In Sweden, dioestrus related diabetes is common in these and other dog breeds, possibly partly explained by the low rate of elective castration of female dogs. Adjusting for breed specific risk of diabetes, however, was not associated with attenuation of the shared risk of diabetes within the owner-dog pairs. Although requirements for daily activity differ between dog breeds,^43^ ^44^ no documented association has been found between risk of diabetes and exercise need. It is, however, plausible that dog owners and dogs share frequency and intensity of exercise and that this could potentially constitute an important underlying mechanism in our findings from owner-dog pairs.

A potential partial explanation for our findings could be shared microbiota influencing diabetes risk in both owners and their pets. Dog owners have been found to share skin microbiota with their pets, and ownership of household pets including dogs and cats has been suggested to be associated with differences in the gut flora of the owner.^45^ ^46^ A recent study also reported a large influence of diet on the gut microbiota of dogs.^47^ It is thus possible that shared microbial communities could influence both owner and dog health, and also that shared dietary and physical activity patterns could affect gut microbiota in dog owners and their pets in a similar fashion. However, the evidence for such a relationship is not available and should be further studied.

Moreover, shared exposures to diabetogenic factors in the environment in terms of noise or air pollution, or exposure to endocrine disrupting chemicals, might influence the risk of diabetes in both owners and their dogs. We did not have access to information on environmental pollutants or chemical exposures in our cohort but we noted no attenuation of our estimates when we adjusted for population density in home municipality, which is a proxy for urban or rural dwelling and might represent different levels of pollution. In addition, the lack of an effect of regional circumstances does not support that distance to healthcare facilities has a large influence on the observed estimates.

Lastly, in a high income country such as Sweden, the risk of type 2 diabetes is higher among individuals of a lower socioeconomic class.^48^ We hypothesised that socioeconomic inequalities encompassing lifestyle factors, health literacy, and access to healthcare could help to explain the shared associations between incidence of type 2 diabetes in dog owners and diabetes in their pets. We therefore adjusted for socioeconomic circumstances, including the covariates cohabitation status, education level, and disposable income of the owner, but we observed no attenuation of the estimates. Assuming our variables truly capture the socioeconomic situation of the owners, we therefore consider it unlikely that socioeconomic circumstances constitute a large underlying mechanism for our findings on shared diabetes risk within owner-dog pairs in our study.

In contrast, we could not detect any association between incidence of type 2 diabetes in cat owners and the development of diabetes in their pets, even though the cat diabetes phenotype more closely resembles that of humans with type 2 diabetes than that of dogs with diabetes. Environmental risk factors for diabetes in cats include indoor confinement, being a greedy eater, eating predominantly dry foods, and being overweight.^13^ We are aware of few studies that have investigated shared lifestyle behaviours between cat owners and their pets. A Dutch cross sectional study, which included 36 owner-cat pairs, reported no association between objectively measured and calculated body mass index of the owner and veterinary assessed body composition of the cats.^18^ One explanation for the lack of association between diabetes in a cat and an owner with type 2 diabetes could furthermore be the lower concordance between cat owner and cat physical activity than between dog owner and dog physical activity.^49^ ^50^ Under the Swedish Animal Welfare Act,^38^ cats who are let outside do not require supervision and cats can even be confined indoors, whereas dogs have to be taken outside at least every six hours during the day for physical exercise. In summary, owner-cat pairs might share fewer health behaviours with regards to dietary habits and physical activity than owner-dog pairs, which could help explain the absence of a shared diabetes risk in owner-cat pairs. This aligns with a recent study that applied a dog ownership relationship scale to cat ownership and found that although emotional closeness and perceived costs were similar between these types of pets, everyday interactions of physical activity and travelling with a pet were more common for owner-dog pairs.^51^ Our owner-cat study population was also substantially smaller than our owner-dog study population. It is therefore also possible that an association between type 2 diabetes in cat owners and diabetes in cats might have gone undetected in our study.

### Conclusions

Owning a dog with a diagnosis of diabetes was associated with an increased hazard of type 2 diabetes in the owner. Potential underlying mechanisms for our findings on diabetes in owner-dog pairs possibly include shared health behaviours such as level of physical activity, and possibly also shared environmental exposures.

**What is already known on this topic**

Dog owners and their pets might share certain health behaviours, such as physical activity level
Cross sectional studies have indicated an association between adiposity in dog owners and their pets
No previous study has investigated shared diabetes risk in dog and cat owners and their pets

**What this study adds**

Dog owners who have a pet with diabetes were more likely to develop type 2 diabetes during follow-up than owners of a dog without diabetes
Personal and socioeconomic circumstances of the dog owners could not help to explain the shared diabetes risk of the owner-dog pairs; underlying mechanisms might include shared diabetogenic health behaviours and environmental exposures
No shared risk of diabetes was found between cat owners and their pets

## Web extra

Extra material supplied by authors

## Notes

Contributors: TF, UH, BK, and RAD designed the study. TF acquired the data, MM cleaned the data, and UH ran the statistical analyses. RAD conducted the literature review and wrote the first draft of the manuscript. TF, UH, BK, MM, RAD, AE, CW, and LB interpreted the results and critically revised the manuscript. TF and BK are the study supervisors and share senior authorship. TF and BK had full access to all the data in the study and are the guarantors. The corresponding author attests that all listed authors meet authorship criteria and that no others meeting the criteria have been omitted.

Ethical approval: This study was approved by the Swedish Ethical Review Authority, Stockholm, Sweden (DNR 2012/1114-31/2, with amendments 2013-1687-32 and 2016/1392-31/1). All analyses were performed on pseudonymised data.

Data sharing: The register data in this study originate from Statistics Sweden, the National Board of Health and Welfare, and Agria Pet Insurance. Restrictions apply to the availability of these data, which were used under license and ethical approval and are not publicly available. Data are, however, available from the authors upon reasonable request and with written permission from the Swedish Ethical Review Authority.

The lead authors (the manuscript’s guarantors TF and BK) affirm that the manuscript is an honest, accurate, and transparent account of the study being reported; that no important aspects of the study have been omitted; and that any discrepancies from the study as planned have been explained.

Dissemination to participants and related patient and public communities: The study population was generated by register linkages between information from Agria Pet Insurance and official Swedish registers. Only pseudonymised data were delivered to the researchers. Direct dissemination of study results to study participants is therefore not possible. No patient organisation is invested in shared diabetes risk of pet owners and their pets.

Provenance and peer review: Not commissioned; externally peer reviewed.
