# Supplementary material for: The shared risk of diabetes between dog and cat owners and their pets: register based cohort study
Source: BMJ. 2020 Dec 10;371:m4337. doi: 10.1136/bmj.m4337 (PMC7726310; doi:10.1136/bmj.m4337)
Supplement: Supplementary file 1 — Supplementary information: additional tables 1 and 2 and figures 1-5 [file delr059840.ww.pdf]

The shared risk of diabetes between dog and cat owners and their pets: register based cohort study

SUPPLEMENTARY MATERIAL

Supplementary table 1. Dog breeds categorized* according to diabetes risk.

	Breeds
**High risk of diabetes**	Australian Terrier, Samoyed, Swedish Lapphund, Swedish Elkhound, Border Collie, Finnish Hound, Drever, West Highland White Terrier, Hamilton Hound, and Poodle (Miniature and Toy).
**Low risk of diabetes**	Jack Russell Terrier, Miniature Dachshund, German Shepherd, Rough Haired Collie, Standard Poodle, Soft Coated Wheaten Terrier, Bearded Collie, Golden Retriever, Boxer, and Papillon.
**Moderate risk of diabetes**	All other pure-breeds as well as mixed-breeds.

* Fall T, Hamlin HH, Hedhammar A, et al. Diabetes mellitus in a population of 180,000 insured dogs: incidence, survival, and breed distribution. J Vet Intern Med 2007;21(6):1209-16. doi: 10.1892/07-021.1 [published Online First: 2008/01/17]

Supplementary table 2. Cat breeds categorized* according to diabetes risk.

	Breeds
**High risk of diabetes**	Burmese, Russian Blue, Norwegian Forest cat, and European Shorthair.
**Low risk of diabetes**	Maine Coon, Persian/Exotic, British Shorthair, Siberian, Birman, Ragdoll, and Bengal.
**Moderate risk of diabetes[1]**	All other pure-breeds as well as domestic cats.

* Öhlund M, Fall T, Strom Holst B, et al. Incidence of Diabetes Mellitus in Insured Swedish Cats in Relation to Age, Breed and Sex. J Vet Intern Med 2015;29(5):1342-7. doi: 10.1111/jvim.13584 [published Online First: 2015/07/17]

[1] Note, that in the article by Öhlund et al, 2015, the cat breed group defined as "Unknown/mix" represented a group of cats with low risk of diabetes. Due to difficulties disentangling this breed group from the domestic cats, it is included in the domestic cat group in our study.

Supplementary Figure 1. Hazard ratios and 95% confidence intervals for type 2 diabetes in dog owners and diabetes in dogs during follow-up from 1 January 2007 to 31 December 2012, illustrating adding of covariates one by one in main analyses. N = 208 980 owner-dog pairs.

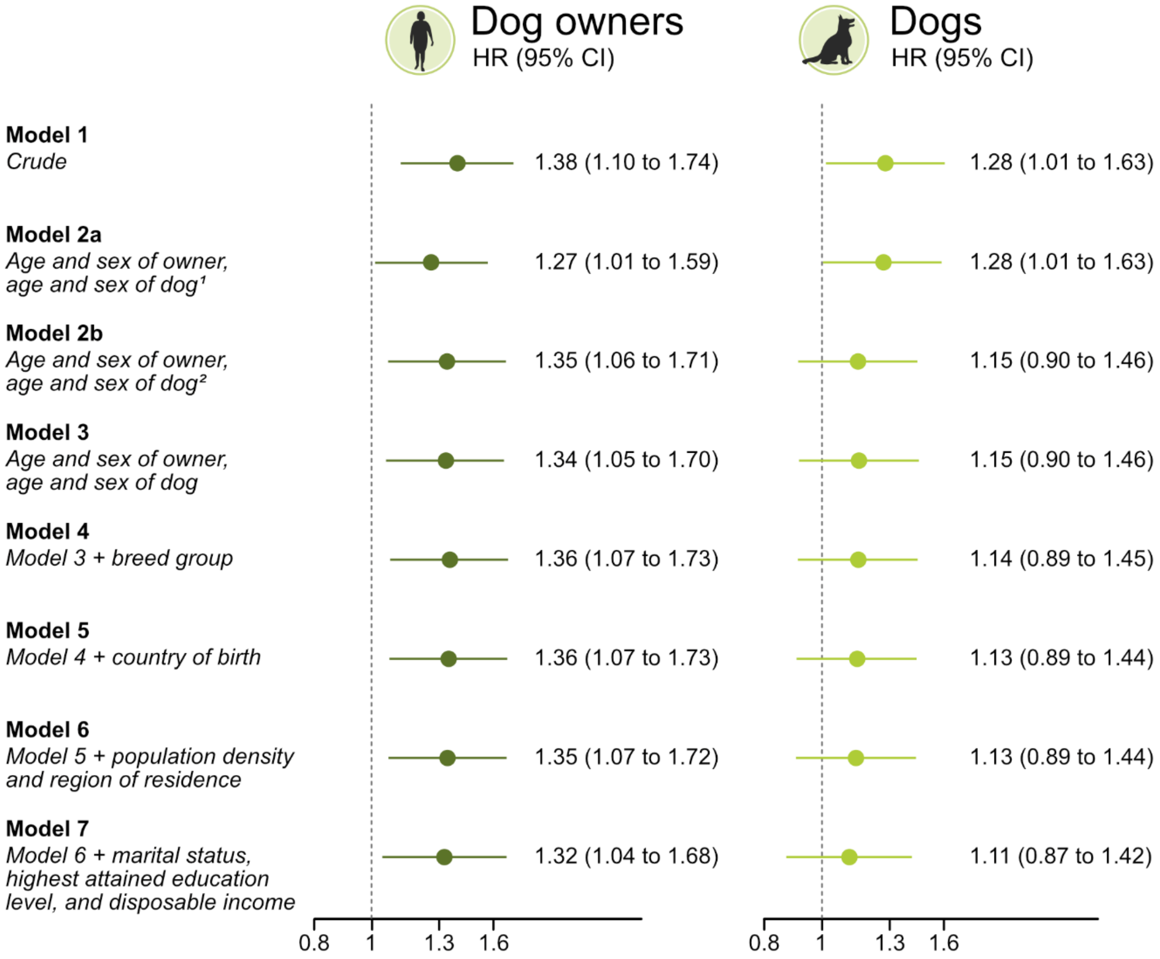

Model 1 corresponds to Crude model in main manuscript, while Model 7 corresponds to Fully adjusted model (Figure 4).

HR: Hazard ratio

CI: Confidence interval

[1] The effect of sex and age of owner on pet's diabetes risk was set to 0, and the effect of sex and age of pet on owner's type 2 diabetes risk was set to 0.

[2] The effect of sex of owner on pet's diabetes risk was set to 0, and the effect of sex of pet on owner's type 2 diabetes risk was set to 0.

Supplementary Figure 2. Association between age of owner and dog diabetes in Model 2b in analyses where covariates were added one by one.

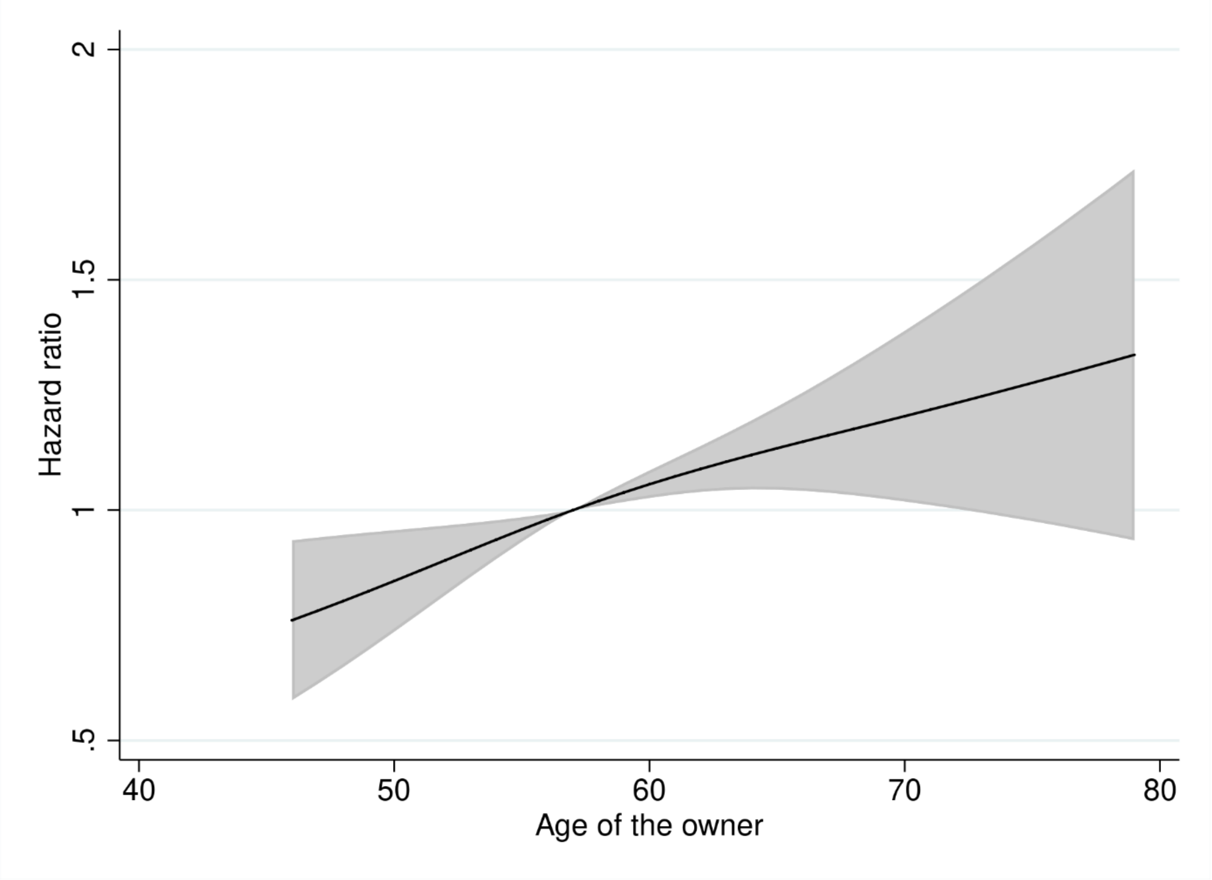

Supplementary Figure 3. Hazard ratios and 95% confidence intervals for type 2 diabetes in cat owners and diabetes in cats during follow-up from 1 January 2007 to 31 December 2012, illustrating adding of covariates one by one in main analyses. N = 123 566 owner-cat pairs

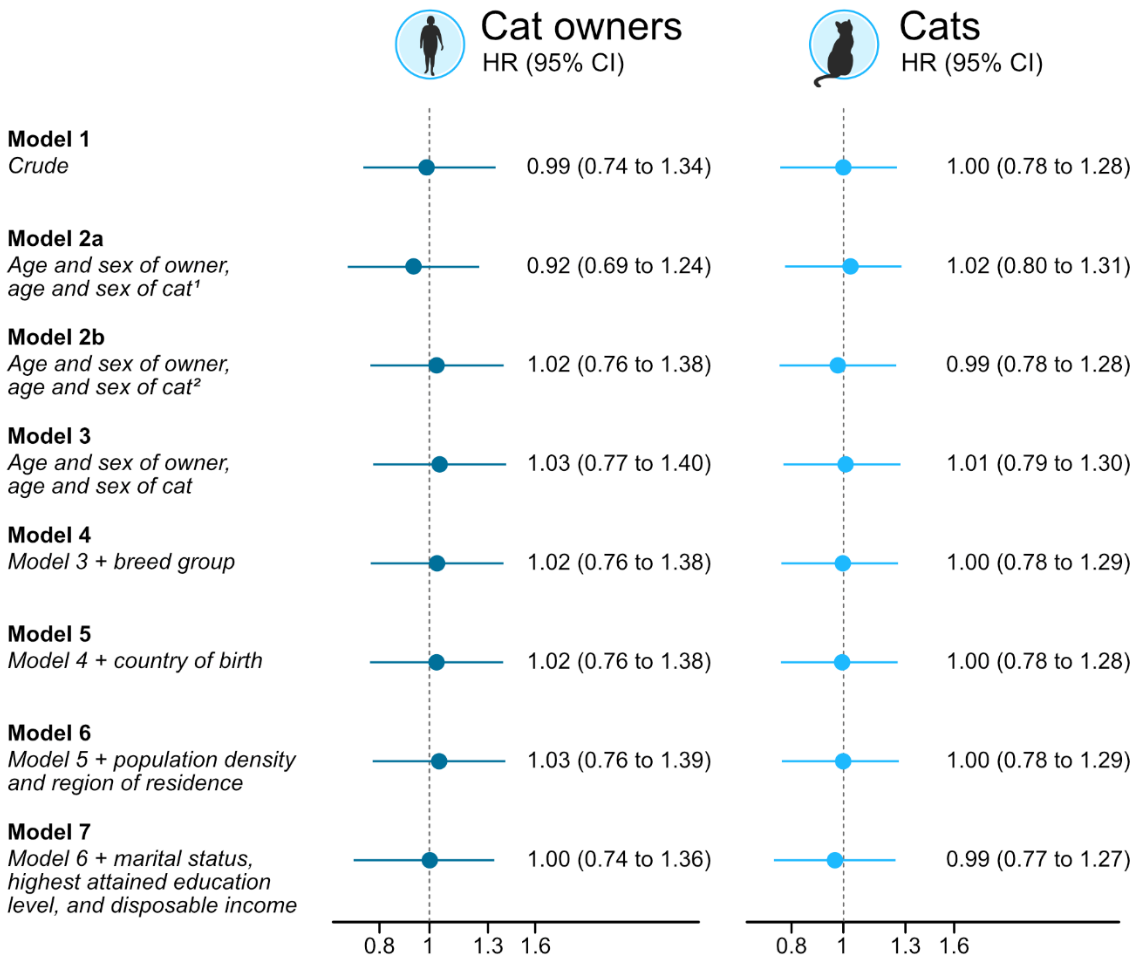

Model 1 corresponds to Crude model in main manuscript, while Model 7 corresponds to Fully adjusted model (Figure 4).

HR: Hazard ratio

CI: Confidence interval

[1] The effect of sex and age of owner on pet's diabetes risk was set to 0, and the effect of sex and age of pet on owner's type 2 diabetes risk was set to 0.
[2] The effect of sex of owner on pet's diabetes risk was set to 0, and the effect of sex of pet on owner's type 2 diabetes risk was set to 0.

Supplementary Figure 4. Sensitivity analysis of risk of type 2 diabetes in dog owners, and risk of diabetes in dogs, respectively, excluding all dog-owner pairs including a dog who had died during baseline or follow-up, regardless of its diabetes status. N = 208 414 owner-dog pairs.

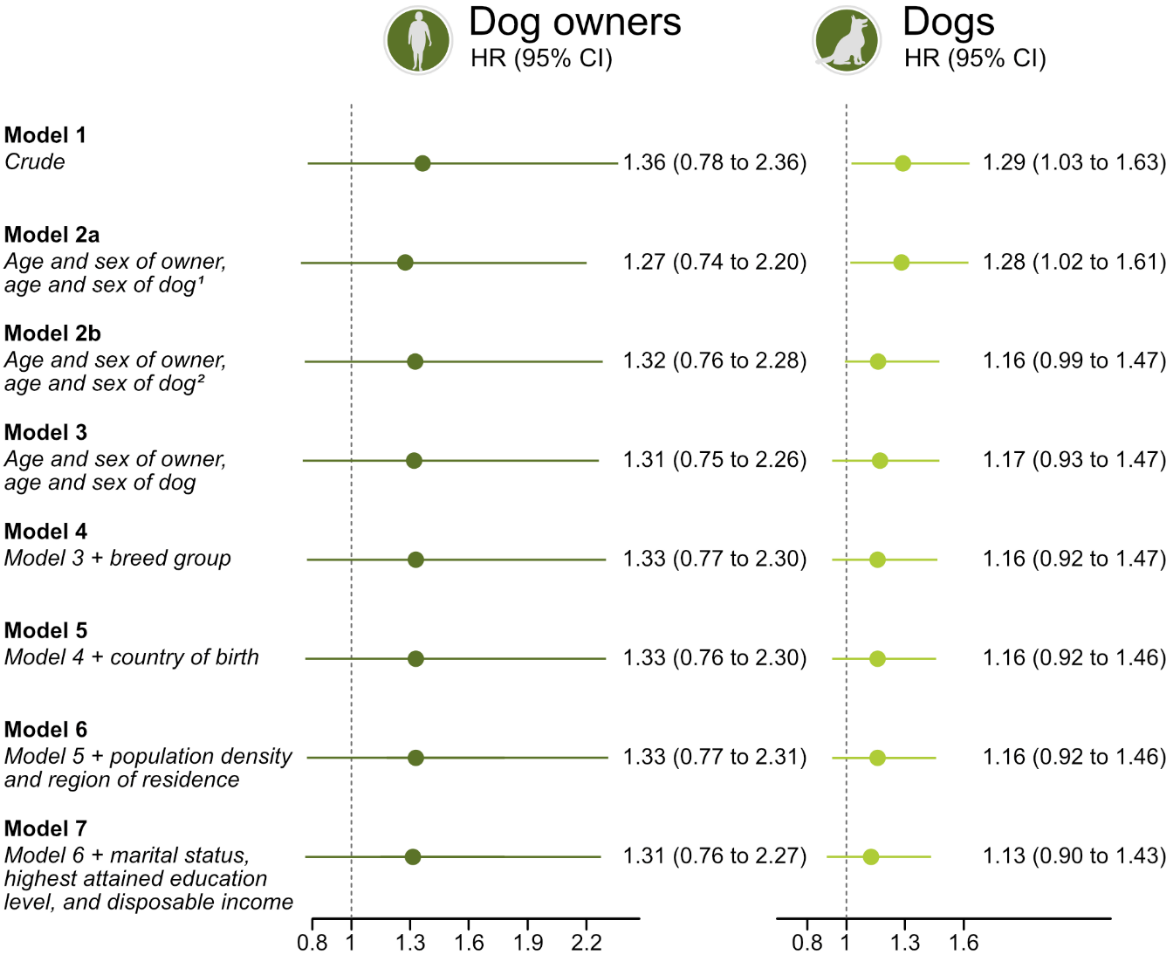

HR: Hazard ratio CI: confidence interval

[1] The effect of sex and age of owner on pet diabetes risk was set to 0, and the effect of sex and age of pet on owner type 2 diabetes risk was set to 0.

[2] The effect of sex of owner on pet diabetes risk was set to 0, and the effect of sex of pet on owner type 2 diabetes risk was set to 0.

Supplementary Figure 5. Sensitivity analysis of risk of type 2 diabetes in cat owners, and risk of diabetes in cat, respectively, excluding all cat-owner pairs including a cat who had died during baseline or follow-up, regardless of its diabetes status. N = 123 232 owner-cat pairs

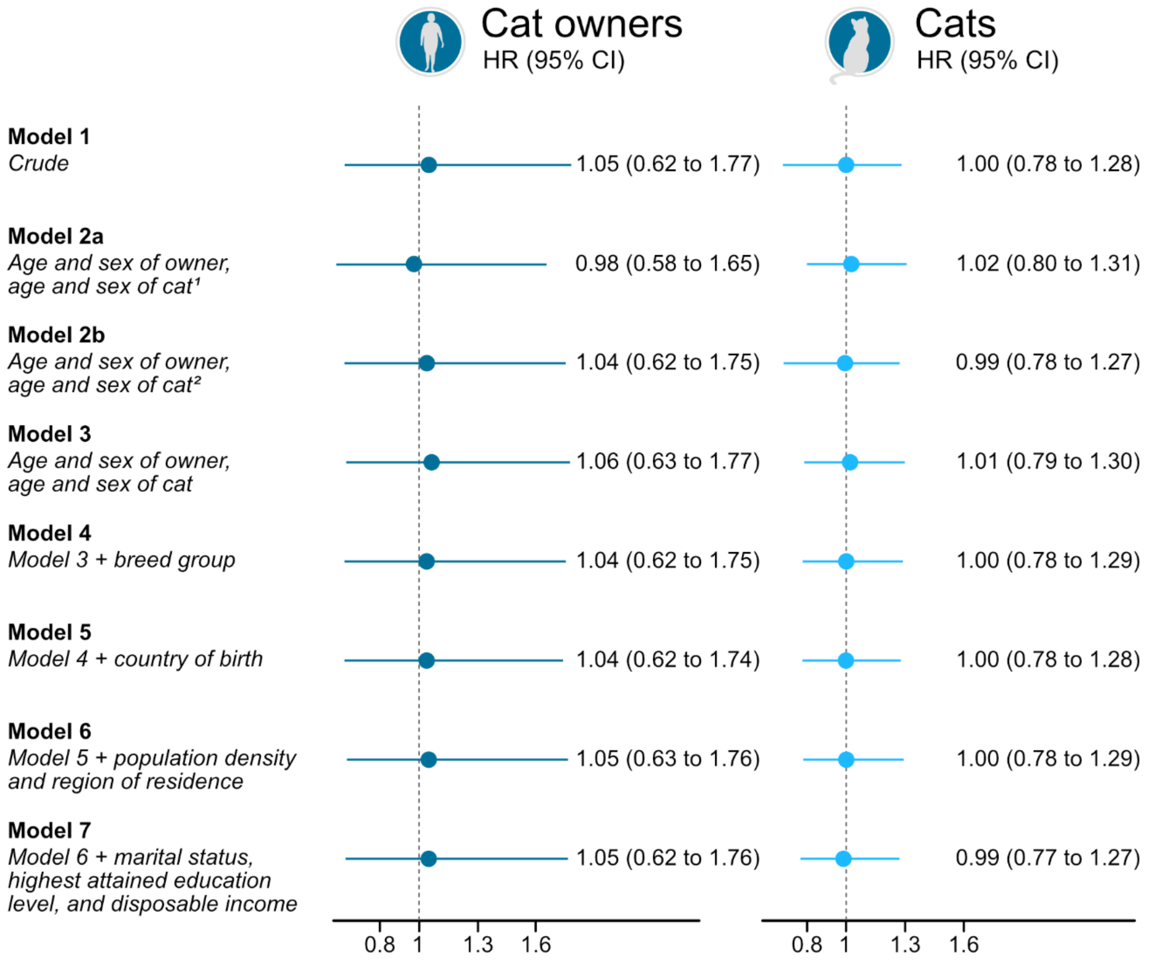

HR: Hazard ratio CI: confidence interval

[1] The effect of sex and age of owner on pet diabetes risk was set to 0, and the effect of sex and age of pet on owner type 2 diabetes risk was set to 0.

[2] The effect of sex of owner on pet diabetes risk was set to 0, and the effect of sex of pet on owner type 2 diabetes risk was set to 0.
